# The role of the cartilaginous to osseous acetabular angle ratio in children with developmental dysplasia of the hip

**DOI:** 10.3389/fped.2024.1347556

**Published:** 2024-03-26

**Authors:** Jiaqi Wang, Tianyou Li, Yangyang Yao, Chaoqun Lu, Yanzhou Wang

**Affiliations:** ^1^ Department of Pediatric Orthopedics, Shandong Provincial Hospital Affiliated to Shandong First Medical University, Jinan, Shandong, China; ^2^ Department of Radiology, Shandong Public Health Clinical Center, Jinan, Shandong, China

**Keywords:** developmental hip dysplasia, acetabular angle ratio, children, MRI, DDH

## Abstract

**Purpose:**

This study aims to demonstrate the use of the cartilaginous to osseous acetabular angle ratio (AAR) in surgical decision-making for hip dysplasia.

**Methods:**

Data were collected from patients who underwent an MRI of the hip after conservative treatment for developmental dysplasia of the hip between August 2019 and 2022. The data included demographic information as well as an anteroposterior pelvic radiograph. The osseous acetabular index (OAI) was measured using x-ray, while the cartilaginous acetabular index (CAI) and the cartilaginous acetabulum head index (CAHI) were measured using MRI. The square of the CAI to OAI, AAR, was calculated. The patients in the residual hip dysplasia (RHD) group were categorized as having an OAI above 20°. During the postoperative follow-up, we evaluated the patients in this group who underwent Bernese triple pelvic osteotomy. Data on surgical patients with an observation period that exceeded 1 year were collected and analyzed. The distribution of the AAR among the different groups was analyzed. A receiver operating characteristic (ROC) predictive model was constructed using the AAR of the patients in the normal and surgical groups to evaluate the need for surgery.

**Results:**

It was found that there was a significant difference in the OAI, CAI, CAHI, and AAR between the RHD group (OAI 26.15 ± 3.90°, CAI 11.71 ± 4.70°, CAHI 79.75 ± 6.27%, and AAR 5.88 ± 4.24) and the control group patients (OAI 16.77 ± 5.39°, CAI 6.16 ± 3.13°, CAHI 85.05 ± 4.91%, and AAR 2.71 ± 2.08) (*p* < 0.001). A total of 93.5% of the control group patients had an AAR ≤5, while only 6.5% had an AAR >5. The results of postoperative imaging follow-up were “excellent” in 52 patients and “good” in 3, while the functional follow-up results were excellent in 53 and good in 2. In 15 patients, the observation period exceeded 1 year. The mean observation period was 633.1 ± 259.6 days and the preoperative CAHI was 71.7 ± 4.8%. Of the patients with an AAR >5, a substantial 94.8% (55/58) of them were reported to have undergone surgery, while all patients with an AAR less than or equal to 5 did not undergo surgery (91/91). Based on the ROC, a cutoff value of 5.09 was identified for the need for surgery in children with RHD.

**Conclusions:**

A surgical decision for residual hip dysplasia can be based on the AAR. An AAR >5 may be a potential indicator for surgical intervention in patients with RHD.

## 1 Introduction

Developmental dysplasia of the hip (DDH) is a congenital musculoskeletal disorder commonly found in children (1). Despite early treatment, residual hip dysplasia (RHD) occurs in a significant proportion of patients, with a rate of prevalence ranging from 3.5% to 17% (2, 3). In addition, it has been identified as the leading cause of early-onset degenerative osteoarthritis. The osseous acetabular index (OAI) is commonly used to assess acetabular dysplasia and determine the necessity of surgery (4). RHD is generally characterized by an acetabular index >20° (5). However, it is widely acknowledged that osseous development of the acetabulum may not accurately reflect acetabular development after bone maturation. Therefore, an accurate evaluation of the cartilaginous development of the acetabulum is crucial (6, 7). The cartilaginous acetabular index (CAI) fully forms at birth and extends almost to the bony acetabulum in adulthood. It remains constant throughout childhood (5). According to several studies, the normal value of the CAI is ≤10° (8, 9). Despite the clinical awareness of acetabular development and the initiation of DDH treatment, a significant proportion of patients with RHD continue to exist. Merckaert et al. reported that the acetabular angle ratio (AAR) could potentially serve as a predictor of acetabular development and surgical intervention and defined its threshold as 5. However, it is worth noting that his study had only a small sample size for analysis and lacked a surgical intervention group or follow-up (9). Against this background, the purpose of this study is to retrospectively analyze data from children with RHD in both operated and non-operated groups, aiming to predict and guide surgical treatment for RHD.

## 2 Materials and methods

### 2.1 Retrospective analysis of patients

A retrospective study was conducted on children who underwent an MRI of the hip after conservative treatment for DDH between August 2019 and August 2022. The inclusion criteria were as follows: patients aged 4–14 years with a previous conservative treatment for hip dislocation or hip dysplasia (including Pavlik harness and closed reduction), with good matching of the hip head and acetabulum (confirmed by the abductive internally rotation position of a pelvic x-ray); this conservative treatment is referenced to Bian (10). The exclusion criteria were as follows: hip subluxation, femoral deformity (femoral head deformity, femoral head necrosis, and flat and short hip deformity), Perthes’ disease, cerebral palsy, and metabolic disorders such as mucopolysaccharidosis and mucolipidosis. Data were collected from a cohort of 152 patients. 44 patients were present in both groups; in group 1, 37 were bilateral and 31 were present in only group 1. In group 2, in addition to the 44 in group 1, another 7 were included in group 2 bilaterally and 33 were included in group 2 unilaterally. The cohort comprised of 25 males and 127 females, with an average age of 83.1 ± 26.8 months.

### 2.2 Measurement of x-ray and MRI images

**Figure 1:**
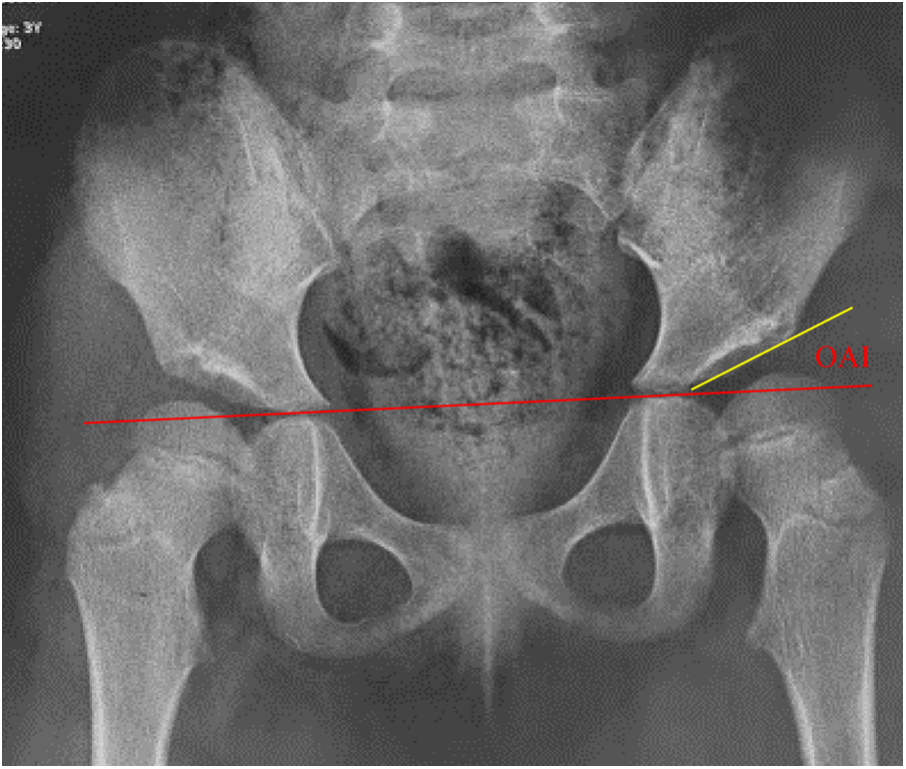
Measurement of the OAI in children with hip dysplasia.

**Figure 2:**
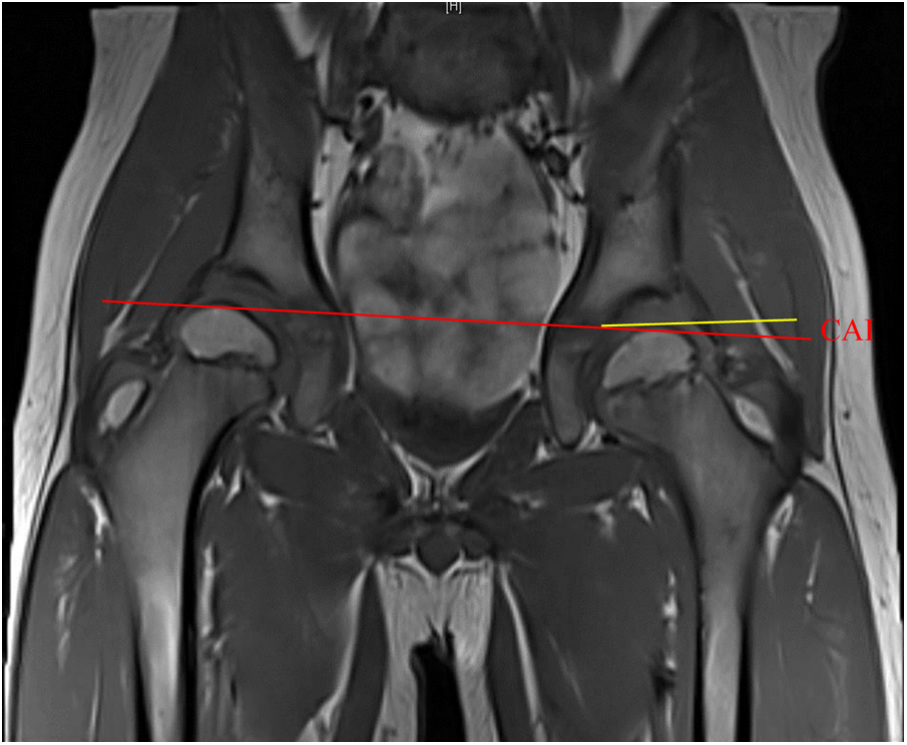
Measurement of the CAI in children with hip dysplasia.

**Figure 3:**
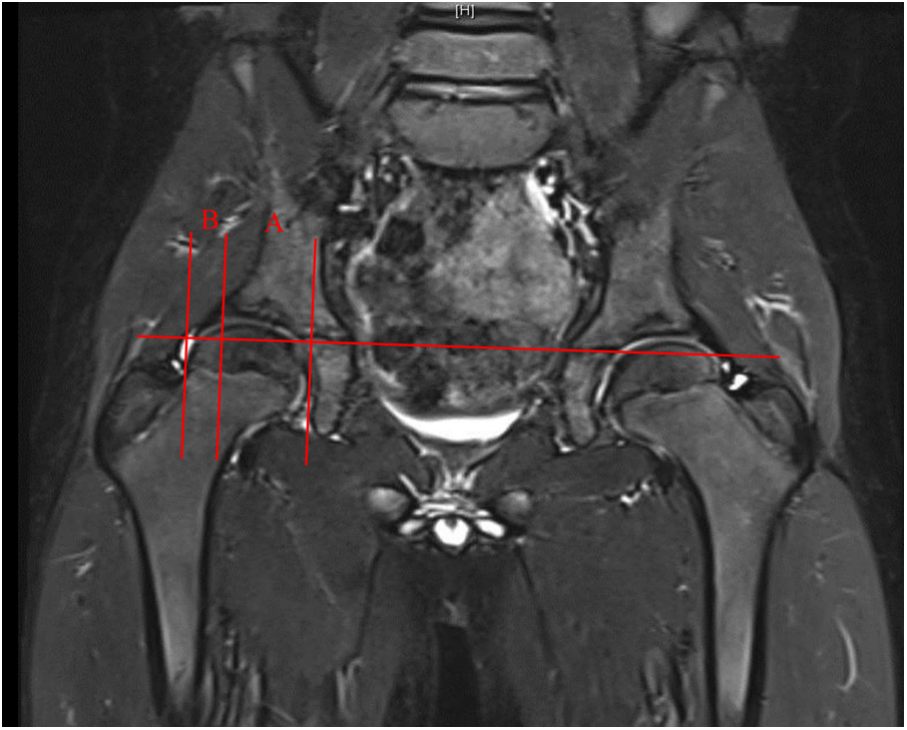
Measurement of the CAHI (*A*/(*A* + *B*)) in children with hip dysplasia.

The OAI was determined by measuring the angle between the line connecting the inferior edge of the ilium and the lateral edge of the bony acetabulum, and Hilgenreiner's line, as illustrated in Figure 1. The CAI was measured as the maximum diameter of the femoral head in the coronal plane of the T1-weighted image, the inferior edge of the ilium and the lateral edge of the cartilaginous acetabulum, and the Hilgenreiner's line, as shown in Figure 2. The AAR was calculated as the value of the square of the CAI to OAI. The cartilaginous acetabulum head index (CAHI) was used to evaluate the lateral cartilaginous acetabular coverage of the femoral head. The measurement of CAHI has been mentioned by Nakamura et al. (11). The results of Sales de Gauzy et al. showed that the range of the CAHI for a normal hip is 77%–93%, and if the CAHI is <77%, it represents inadequate coverage of the femoral head by the acetabulum (12). We conducted measurements in T2 images for the purpose of better identification of cartilage borders. The coronal images of the maximum diameter of the femoral head were obtained on a T2-weighted image, and the distance from the innermost edge of the femoral head cartilage to the outermost edge of the acetabular cartilage (*A*) and the maximum transverse diameter of the femoral head (*A* + *B*) were measured, and the CAHI = *A*/(A + *B*) × 100% as shown in Figure 3. The normal value of the OAI at birth is below 30°, reducing rapidly in the first 4 years toward 15 ± 5.5° and remaining stable until full hip ossification at maturity (13–15). We defined patients with RHD as having an OAI > 20° on the x-ray, as described by Merckaert et al. (9), and they were categorized as group 1, the residual hip dysplasia group, while healthy patients were categorized as group 2, the control group. The data of the two groups, as well as the distribution of the AAR in each population, were statistically analyzed.

### 2.3 Follow-up of postoperative patients

The patients in group 1 who underwent the Bernese triple pelvic osteotomy were subjected to imaging and functional follow-ups. The follow-up time was calculated as the period between the date of the patient's surgery and the final follow-up time. All surgical patients had a mean follow-up time of 26.7 ± 9.0 months. The x-rays of the patients at the end of the follow-up period were obtained, and the OAI and center edge (CE) angles were measured for imaging evaluation according to the modified Severin classification (16). The follow-up of hip function was performed according to the modified McKay criteria (17). In this subgroup of patients who underwent surgery, the period between the diagnosis of RHD and the time of surgery was defined as the observation period. Data on the patients who underwent surgery with an observation period of more than one year were collected and their preoperative parameters analyzed. In addition, a comparison of the AAR of surgical patients with that of non-surgical patients was performed. Since the surgical patients showed satisfactory follow-up results, we initially identified this subgroup of patients as those who needed surgery.

### 2.4 Statistical methods

The data collected were analyzed using SPSS 26.0 software. The mean ± standard deviation (SD) was used to represent normally distributed data. Comparisons between groups were made using independent sample t-tests. The receiver operating characteristic curve (ROC) curve was constructed with their AAR vs. group 2. We also evaluated the significance of the AAR do decide whether to perform surgery. A *p*-value of less than 0.05 was considered statistically significant.

## 3 Results

The study included 149 hips of patients in group 1, with a mean OAI of 26.15 ± 3.90°, a mean CAI of 11.71 ± 4.70°, a mean CAHI of 79.75 ± 6.27%, and a mean AAR of 5.88 ± 4.24. Group 2 consisted of 91 hips of patients, with a mean OAI of 16.77 ± 5.39°, a mean CAI of 6.16 ± 3.13°, a mean CAHI of 85.05 ± 4.91%, and a mean AAR of 2.71 ± 2.08. The results are presented in Table 1.

**Table 1:** Statistical results of group 1 and group 2 of OAI, CAI, CAHI and AAR.

	Group 1	Group 2	*p*-value
Total (hip)	149	91	—
OAI (degrees)	26.15 ± 3.90	16.77 ± 5.39	<0.001
CAI (degrees)	11.71 ± 4.70	6.16 ± 3.13	<0.001
CAHI (%)	79.75 ± 6.27	85.05 ± 4.91	<0.001
AAR	5.88 ± 4.24	2.71 ± 2.08	<0.001

The independent sample t-test comparing the OAI, CAI, CAHI, and AAR in group 1 and group 2 showed a significant difference in imaging parameters between the normal and the residual acetabular dysplasia group of patients (*p* < 0.001). An analysis of the AAR distribution pattern showed that 93.5% of group 2 patients had an AAR ≤ 5, while only 6.5% had an AAR >5.

The preoperative CAHI of 55 patients was 75.0 ± 5.2%. The postoperative OAI was measured at a mean of 13.4 ± 5.7° and a CE angle of 29.7 ± 6.0°, and imaging follow-up was done according to the modified Severin classification; the results were “excellent” in 52 patients and “good” in 3. No limp or hip pain was reported and the hip showed good mobility. Functional assessment was performed according to the modified McKay criteria, and the results were excellent in 53 patients and good in 2. The observation period exceeded 1 year in 15 patients, with a mean of 633.1 ± 259.6 days. The preoperative CAHI was 71.7 ± 4.8%.

Surgical patients accounted for 94.8% (55/58) of those with an AAR >5% and 0% of patients with an AAR ≤5. On the other hand, non-surgical patients accounted for 100% (91/91) of those with an AAR ≤5. Three non-surgical patients with an AAR >5 refused treatment, and therefore were not followed up. The results are presented in Table 2.

**Table 2:** Distribution of hips with AAR greater than 5 versus less than 5 and with or without surgery in group 1.

	AAR ≤ 5	AAR > 5
Operated	0	55
Non-operated	91	3

**Figure 4:**
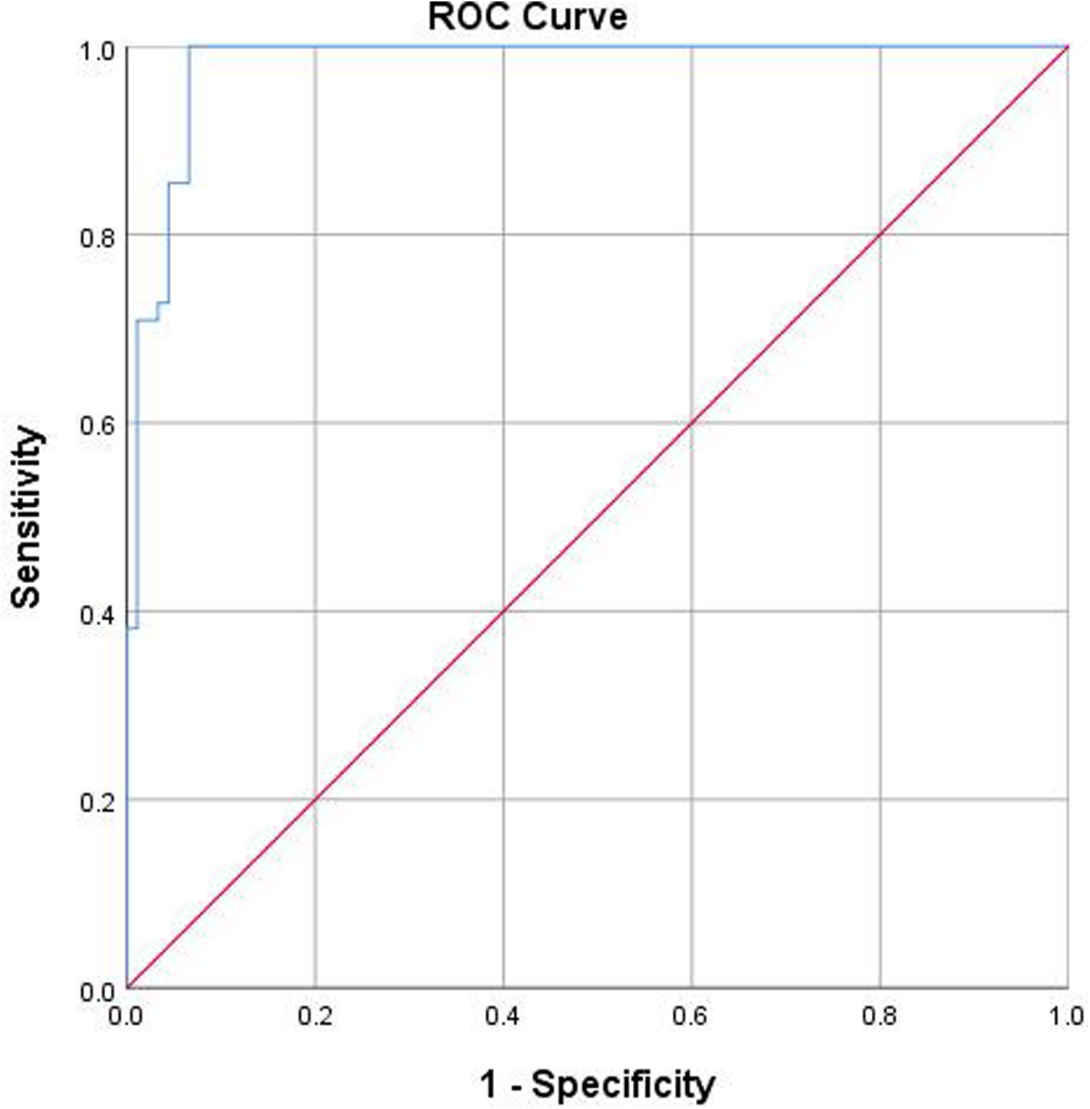
ROC for the necessity of surgery in children with DDH diagnosed by the AAR.

In the ROC analysis, the area under the curve was 0.981, the standard error was 0.009, *p* < 0.01, the 95% confidence interval was 0.962–0.999, and the cutoff value was 5.09, which suggests that using the AAR as an indicator for surgery in children with RHD is a sensitive approach, as shown in Figure 4 and Table 3.

**Table 3:** Area and 95% confidence interval of AAR as an indicator for surgery in children with RHD.

Area under the curve				
Test result variable(s): AAR				
Area	Std. error^a^	Asymptotic sig.^b^	Asymptotic 95% confidence interval	
			Lower bound	Upper bound
0.981	0.009	0.000	0.962	0.999

^a^ Under the non-parametric assumption.

^b^ Null hypothesis: true area = 0.5.

## 4 Discussion

The treatment of DDH is a complex procedure that involves various aspects such as the acetabular and femoral structures, the soft tissues, and their interrelationships. Age is also an important factor to consider. Initially, x-ray was used as a diagnostic and treatment tool for DDH. It is a quick and easily accessible imaging tool; however, it comes with the drawback of radiation exposure and difficulties in evaluating the soft tissue portion of the hip joint. In contrast, MRI is a fast, non-invasive, highly sensitive, and specific examination that does not involve radiation like x-rays or CT scans. It offers a wide range of information about the femoral head, acetabulum, and labrum. MRI has proven to be advantageous in demonstrating bone, cartilage, and soft tissue structures (18, 19). Previously, the CAI was mainly measured on T2-weighted images (11), in which the morphology of the labrum and any damage could also be clearly observed. However, it is easier to differentiate between osseous and cartilaginous acetabular structures using T1-weighted images. Hip dysplasia is characterized by an abnormality in the relationship between the femoral head and the acetabulum (20). Some studies recommend secondary surgery for the treatment of RHD before the age of 5 or 6 based on radiographic evaluation (21, 22). However, predicting acetabular remodeling solely based on radiographic evaluation is challenging. In addition to evaluating the osseous acetabulum, it is important to assess the cartilaginous acetabulum in order to predict future acetabular development. The timing of surgery in patients with RHD is still a matter of debate (23, 24).

Fisher et al. first suggested the use of MRI to measure the OAI vs. CAI in order to assess osseous and cartilaginous coverage. They discovered that all hips with an OAI >30° and a CAI >10° exhibited varying degrees of dysplasia (25). Walbron et al. conducted a study to determine adequate acetabular coverage and found that an OAI of <18° on radiographs was considered sufficient (26). In a similar study, Huber et al. (8) analyzed 115 normal hips of 73 children using MRI and found that the CAI remained consistent during growth, with a mean CAI of 5°. In addition, approximately 90% of the CAI measurements remained below 10°, suggesting that hips beyond of this range could be classified as RHD. In our study, the control group exhibited a mean CAI of 6.16° ± 3.13°, which falls within the normal range of the CAI. In line with the findings of Huber et al. and Li LY, we also define hip dysplasia as an OAI <20° (5, 8). Furthermore, the measurements of the OAI on MRI correlate with those of the OAI on plain radiographs (5). Therefore, it became more convenient for us to measure the OAI on x-rays.

Merckaert et al. reported that the AAR can serve as a predictor of acetabular development and surgical intervention. This study marks the first instance in which the AAR has been proposed as an indicator of the trend of development in the cartilaginous acetabulum (9). In our own research, we observed a notable disparity in the AAR between patients in group 1 and group 2, with a *p*-value <0.05. Within the subgroup of residual acetabular dysplasia, we discovered a substantial proportion of patients exhibiting favorable cartilage development and head coverage. Specifically, 91 patients (61.1%) displayed these positive outcomes, as demonstrated by their AAR measurements, all of which were <5. Although this subset of patients was diagnosed with dysplasia on x-ray, they showed a favorable trend of cartilage development. Therefore, if their cartilaginous acetabulum continues to develop as expected, then they do not require subsequent surgical intervention. Fifteen patients remained poorly covered with cartilage after an observation period of more than 1 year, and this group of patients may not initially require continued observation. Since the AAR is >5 in this group of patients, it means that their cartilage is insufficiently developed, and this congenital insufficiency is not a favorable trend for them even after observation. Therefore, when we find patients with RHD, we need to perform MRI to evaluate their cartilage acetabular development and calculate the AAR, so as to predict their future development trend and whether they need surgical intervention at a later stage.

**Figure 5:**
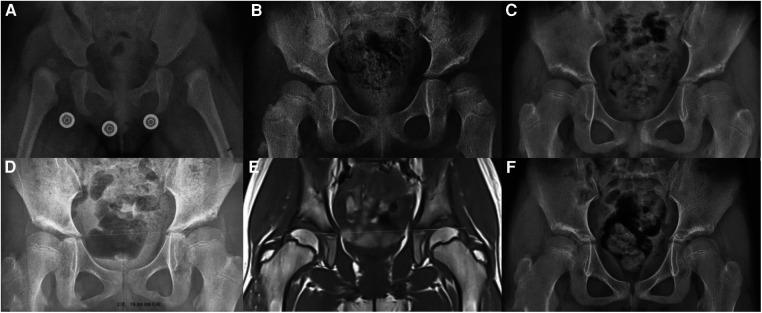
(**A**) A child with a bilateral DDH with bilateral hip dislocation was diagnosed at 6 months and treated conservatively. (**B**) A child 3 years and 7 months of age with developmental dysplasia with residual acetabular dysplasia. (**C**) A child 6 years and 3 months of age. (**D**) A child 7 years and 8 months. (**E**) The same age as (**D**), left CAI 1° with good cartilage coverage (CAHI 85.6%). (**F**) A child 8 years and 7 months of age with a normalized left hip.

**Figure 6:**
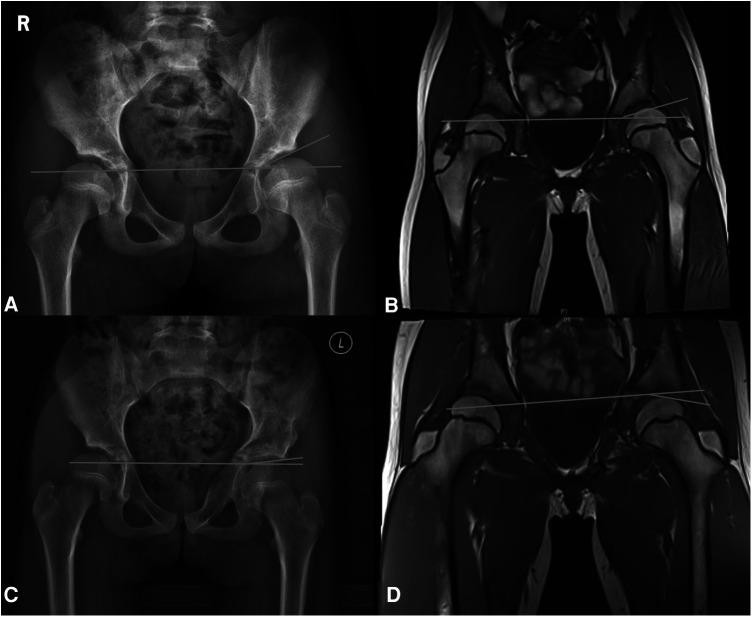
(**A**) A child 9 years old, previously treated conservatively with bilateral RHD. (**B**) An MRI of a 9-year-old child showing a left CAI of 16.1° and an AAR of 10.36. (**C**) An 11-year-old child, 2 years after the performance of Bernese triple pelvic osteotomy on the left side. (**D**) A postoperative MRI of a child with a left CAI of −14.3°.

Figure 5 depicts a patient who experienced bilateral hip dislocation at 6 months of age and received conservative treatment. Regular follow-up revealed dysplasia at 3 years of age, with the left side being more severely affected. At 7 years and 8 months, she had an MRI, the left side was more affected on x-ray than before. The patient was followed up until 8 years and 7 months. A retrospective analysis of the AAR showed a value of 0.06, which meant adequate coverage. Figure 6 presents another case of a child with RHD. As shown in Figure 6A, the child was 9 years old and had previously experienced bilateral DDH and RHD, which were treated conservatively. An x-ray revealed that the left side was more affected. As shown in Figure 6C, the child underwent a left Bernese triple pelvic osteotomy at the age of 9 due to left-side pain. The child is now 11 years old, with a postoperative period of 2 years, and radiographic and functional follow-ups based on the modified Severin classification and the modified McKay criteria indicate excellent results. A retrospective analysis of preoperative and postoperative MRI findings is summarized. As shown in Figure 6B, the preoperative MRI reveals a left CAI of 16.1° and an AAR of 10.36, which are significantly higher than 5. On the other hand, Figure 6D displays the postoperative MRI with a left CAI of −14.3° and a well-covered femoral head. The patient in Figure 5 has a preoperative AAR <5 and does not require surgery. In contrast, the patient in Figure 6 has a preoperative AAR >5 and theoretically should have undergone surgery, which was confirmed by the actual surgical decision taken, highlighting the predictive value of the AAR.

We performed the procedure of Bernese triple pelvic osteotomy in a subset of RHD patients, taking into account the surgeon's experience and radiographic findings. A total of 55 children were treated with surgery due to RHD, all of whom had an AAR >5. However, three children were not included in the study as their families refused to give consent for surgery, and therefore, these children were not followed up. We achieved satisfactory imaging and functional outcomes in all patients who underwent surgery, which aligns with the findings of Merckaert et al. (9). Their research indicated that children with an AAR >5 required surgical intervention, whereas those with an AAR <5 exhibited adequate cartilage coverage and therefore did not require surgery. Moreover, we conducted an in-depth analysis of the AAR results between the control and the surgical groups. Through predictive modeling and an ROC analysis, we determined a cutoff value of 5.09. This value serves as a potential indicator for surgical intervention in patients with dysplasia. In the future, we plan to conduct a prospective study using an AAR >5 as a reference for determining the need for surgery. Furthermore, we discovered instances of an AAR >5 in patients with normal hips. For example, one patient exhibited an OAI of 13°, a CAI of 9°, and a calculated AAR of 6.23. Another patient showed an OAI of 16°, a CAI of 10°, and an AAR of 6.25. However, it was relatively easy to identify and exclude these patients based on their history and radiographs recorded during clinic visits. It was possible that these irregularities were a result of systematic errors.

There have been reports of studies conducted using the cartilaginous center edge (CCE) angle as a reliable imaging index for evaluating hip dysplasia and prognosis. In a retrospective study by Takeuchi et al. (27), 119 patients with RHD were examined, and it was discovered that if the CCE angle measured >13° on MRI at 2 years of age, it indicated that the acetabulum would develop normally. Similarly, Nakamura et al. (11) conducted a retrospective evaluation of 92 patients with acetabular dysplasia without a history of DDH to assess the role of MRI in predicting regression. If the CCE angle measured ≥23°, it suggested that the hip would develop appropriately without the need for surgical intervention. However, even with adequate cartilage, normal and complete ossification is not always possible, and Wakabayashi et al. (28) reported that an abnormal loading of the acetabular cartilage could lead to an edematous alteration or dysregulation of proteoglycan distribution, resulting in impaired acetabular cartilage ossification and, ultimately, acetabular dysplasia. Therefore, follow-up is a dynamic monitoring process, and intervention is also necessary when there is an abnormality in the process of cartilage ossification. We suggest that an MRI is still needed at the age of 8 years to assess the presence of abnormal cartilage ossification and the need for surgical intervention in patients with RHD who are being followed up. Although many surgeons currently choose to observe patients with RHD, it is theoretically necessary to determine the development of the cartilaginous acetabulum based on the AAR. If the AAR is >5, there is no need to continue observation, and immediate surgical intervention can be initiated. In this study, we found that 15 patients showed no improvement in acetabular development after more than 1 year of observation, and the retrospective analysis of their cartilage development was insufficient. However, the timing of surgery needs to be determined, as also the patient's age, the risk of surgery, the parents’ wishes, etc. More often, the timing of intervention is chosen after the patient attains 5 years of age (29).

Our study also has some limitations. DDH is a complex disease, and the patient cases we studied were limited to RHD with a well-matched head and acetabulum after conservative treatment, without any deformity of the acetabulum or femur. This represents only a small proportion of DDH cases. In addition, we examined only a small number of patients and the follow-up period was relatively short. Moreover, the measurements of imaging data were limited to only one observer, which did not account for the differences among different observers.

In conclusion, for patients with RHD with a well-matched head and acetabulum, surgical decisions can be made on the basis of the AAR. An AAR >5 may serve as a potential indicator for surgical intervention in RHD patients.

## Data Availability

The raw data supporting the conclusions of this article will be made available by the authors without undue reservation.
